# How Different Are Our Perceptions of Equal-Tempered and Microtonal Intervals? A Behavioural and EEG Survey

**DOI:** 10.1371/journal.pone.0135082

**Published:** 2015-08-18

**Authors:** Freya Bailes, Roger T. Dean, Mary C. Broughton

**Affiliations:** 1 School of Drama, Music & Screen, University of Hull, Hull, United Kingdom; 2 MARCS Institute, University of Western Sydney, Penrith, New South Wales, Australia; 3 School of Music, University of Queensland, Brisbane, Queensland, Australia; University Zurich, SWITZERLAND

## Abstract

For listeners familiar with Western twelve-tone equal-tempered (12-TET) music, a novel microtonal tuning system is expected to present additional processing challenges. We aimed to determine whether this was the case, focusing on the extent to which our perceptions can be considered bottom-up (psychoacoustic and primarily perceptual) and top-down (dependent on familiarity and cognitive processing). We elicited both overt response ratings, and covert event-related potentials (ERPs), so as to compare subjective impressions of sounds with the neurophysiological processing of the acoustic signal. We hypothesised that microtonal intervals are perceived differently from 12-TET intervals, and that the responses of musicians (n = 10) and non-musicians (n = 10) are distinct. Two-note chords were presented comprising 12-TET intervals (consonant and dissonant) or microtonal (quarter tone) intervals, and ERP, subjective roughness ratings, and liking ratings were recorded successively. Musical experience mediated the perception of differences between dissonant and microtone intervals, with non-musicians giving similar ratings for each, and musicians preferring dissonant over the less commonly used microtonal intervals, rating them as less rough. ERP response amplitude was greater for consonant intervals than other intervals. Musical experience interacted with interval type, suggesting that musical expertise facilitates the sensory and perceptual discrimination of microtonal intervals from 12-TET intervals, and an increased ability to categorize such intervals. Non-musicians appear to have perceived microtonal intervals as instances of neighbouring 12-TET intervals.

## Introduction

There has been considerable discussion of the extent to which our perceptions of the pitch and quality of tones, including their place in hierarchical scale systems and tonality, depend not only on their psychoacoustic properties, but also on our familiarity (enculturation) with the system(s) in which they are used [1]. Baldly, these two factors can be considered bottom-up (psychoacoustic and primarily perceptual) and top-down (dependent on familiarity and cognitive processing). Here we wished to address these issues further, and to compare musicians and non-musicians, with both explicit behavioural methods and indirect electroencephalography (EEG) methods, arguing that EEG might reveal features of listeners' responses that are not displayed in explicit measures.

Our aim was to determine whether microtonal intervals separating two simultaneously presented tones are perceived in a different way from equal-tempered intervals. A microtonal system is one that uses intervals other than the logarithmic frequency ratio intervals of Western 12-tone equal temperament (12-TET). In 12-TET, the octave, an interval comprising a frequency ratio of 2, is divided into 12 equal ratio steps (‘semitones’), each of which is a ratio of the 12^th^ root of 2, c. 1.06. Most microtonal tuning systems are N-TET and repeat their pattern every octave. In practice, performed musical intervals in Western (12-TET) music rarely conform to the exactitude of equal temperament, yet they are probably often taken as approximations to equal-tempered categories. There is considerable evidence that intervals can be perceived categorically in some circumstances. In this context, categorical indicates that “signals that vary over a continuous physical scale are perceived as belonging to a small number of discrete groups” [2]. Klein and Zatorre examined the brain activity of musically trained participants associated with the categorical perception of major and minor chords. Their experimental stimuli were composed of intervals ranging in 10-cent increments between minor and major triads (which are 100 cents, or one semitone, different), while their control stimuli were composed of variations in the absolute pitch of triads fixed at a ratio of 350-cents, representing the quarter-tone midpoint between a major and a minor third. Discrimination functions revealed that participants perceived the range of experimental stimuli categorically, but not the control stimuli. Such categorical perception of microtonal intervals is indicative of a degree of top-down influence.

Of further interest in the present work is how perceptions of other qualities of simultaneously presented tones, such as roughness, differ. A sensation of roughness is elicited by dissonant intervals—those whose notes form large-integer fundamental frequency ratios (e.g. tritone 32:45) [3]. Many of the overtones of dissonant intervals fall within what is known as the critical bandwidth (approximating four semitones) and interaction of conflicting overtones creates fast amplitude fluctuations that are unable to be resolved by the basilar membrane in the human auditory system.

It has been known for more than a century (see review and data in Plomp & Levelt [4]) that perceptions of pleasantness of simple pure tone intervals and complex tone intervals are very closely related to those of perceived 'consonance', and largely predicted by the critical bandwidth associated with the lower pitch. The critical bandwidth is the interval which permits maximal consonance in these circumstances, and the maximum unpleasantness is reached at an interval close to 0.25*(critical bandwidth) [4]. Beyond the critical bandwidth (maximum pleasantness and consonance) there is a slight further increase in unpleasantness.

In a preliminary study of the perceptions of 16 non-musicians to microtonal intervals from the 81 sequential primes just tuned scale [5], ratings of pleasantness varied little with frequency difference between the two simultaneously presented tones, apart from showing a substantially elevated value for the 40 Hz frequency difference, which is close to 0.25*(critical bandwidth) across the range of frequencies we studied. Respondents also rated the perceived distance between frequencies in these microtonal intervals, with no indication that pitch distances amongst these microtonal intervals were perceived differently from those described amongst 12-TET intervals. The findings were in keeping with earlier observations on tonal intervals, and the fewer observations on systematic microtonal intervals such as those above. Microtonal intervals were thus not treated uniquely. However, even when there is little explicit behavioural effect of a feature, implicit measures such as EEG may reveal informative differences in perception. Thus in our main experiment we undertook event related potential (ERP) and behavioural measures in tandem.

Music theory relating to 12-TET distinguishes 'perfect consonances', 'imperfect consonances' and 'dissonances' (see [1]), partly because of their conceived role in the composition of harmony in tonal music (music in which a particular pitch can dominate, constituting the key of the current section of the piece). Perfect consonances comprise the intervals of unison, octave (1200 cents), perfect fourth (500 cents), and perfect fifth (700 cents). Dissonances comprise minor seconds (100 cents), major seconds (200 cents), augmented fourths (600 cents), minor sevenths (1000 cents) and major sevenths (1100 cents). Imperfect consonances comprise the remaining four intervals within the octave. We are concerned here with their perceived consonance and pleasantness, and as the names imply, this is generally ordered with perfect intervals being more consonant and pleasant than imperfect intervals, which are perceived to be more consonant and pleasant than dissonant intervals. Broadly, these lines of argument suggest that microtonal intervals might either appear unpleasant, like dissonances, or sometimes be categorically assimilated to the nearest consonance.

In a previous ERP study of sensory consonance, distinctions between perfect and imperfect consonant intervals were found in the 400-600ms latency band of the ERP, and showed a consonance-electrode interaction [6]. Here, imperfect consonances induced larger negativity in centro-parietal regions about 420ms. ERP responses in this time frame are normally more cognitive than perceptual. A reasonable and widely used assumption in the interpretation of ERP signals is that the greater the absolute response, the greater the neural processing involved. In addition, negative and positive peaks in the response are associated with specific aspects of stimulus processing. The earliest parts of the ERP are predominantly perceptual processes, while as time progresses cognitive events contribute more. Schön and colleagues [6] proposed that the N420 observed reflected difficulties in categorising imperfect consonant intervals, and could represent a delayed, yet functionally similar N2 component. Similarly, in the context of roughness, there are also observations of distinctly different neural correlates comparing consonant and dissonant music [7]. It is less clear in the literature what the corresponding effects of microtonal intervals may be, which is one of the purposes of the present work.

### The role of music training in interval perception

It is conceivable that in some circumstances, listeners categorise microtonal intervals into the small number of consonant and dissonant intervals of the 12-TET octave. The question is whether musicians and non-musicians differ in this respect. Instrumental music training appears to induce neuroplasticity, bringing about enhanced sensorimotor and cognitive performance in related tasks [8]. Findings of ERP studies of sensory consonance involving interval or chordal music tones support the notion of training induced neuroplasticity modulating passive as well as explicit responses [9]. Kung et al. [10] played their amateur-musician and non-musician participants perfect fifths and augmented fourths. Musicians showed greater ERP amplitude for augmented fourths at N1 (in this usage, N1 corresponds to a temporal region around 100ms) and N2, and greater P2 amplitude for perfect fifths. When the ERPs were sorted according to roughness (rather than music theory) non-musicians showed greater amplitude for non-rough intervals at P2, and greater amplitude for rough intervals at N2. Itoh, Suwazono, & Nakada [11] found that both P2 and N2 peaks occurred when musician and non-musicians listened to intervals where tones were separated by a semitone (the smallest difference between tones according to 12-TET). The interval effect observed at P2 vanished once intervals separated by up to three semitones were removed from analysis (considered here the ‘critical bandwidth’). This led the authors to conclude that sensory consonance, rather than musical training was the basis for listeners distinguishing intervals at P2. At N2 when the intervals separated by up to three semitones, an interval main effect occurred for musically trained, but not non-musician participants. Furthermore, the musically trained participants’ N2 amplitudes were greatest for dissonant intervals, becoming more positive in amplitude according to the consonance ascribed by Western music theory.

Differences between musician and non-musician ERPs have also been observed at N1, P2 and N2 components in studies combining ERP acquisition and a behavioural task. For example, Minati et al. [9] had musician and non-musician participants listen to equal numbers of consonant and dissonant four-note chords, and categorise each as either consonant or dissonant. P1 was more positive for consonant chords. No significant effects were observed for N1 or P2. N2 showed greater negativity for dissonant chords, and musicians showed a greater chord-type effect. The chord-type effect was not significant for non-musicians at N2. Following the N2 shown by musicians for consonant chords, there appeared a small positive component with a peak around 350ms. Minati et al. [9] proposed that this component could reflect a weak P3, reflecting stimulus recognition (as previously found by [12]).

Somewhat contrary to others [9–11], Schön and colleagues [6] found that non-musicians’ N2 component was significantly more negative for perfect consonances than dissonances and imperfect consonances. Schön et al. [6] also found that musicians’ N1-P2 complex was more positive for very pleasant (cf. unpleasant) intervals—a finding similar to that of Kung et al. [10]. In sum, EEG results indicate a difference in processing 12-TET intervals between musically trained and untrained participants.

In a complementary way, research by Brattico et al. [13] revealed stronger change-related mismatch negativity (MMNm) for musicians than non-musicians when they heard dissonant and mistuned chords in the context of a sequence of major chords. Their study was designed to determine whether musical expertise is associated with generally superior auditory discrimination, or enhanced differentiation of culture-specific music categories, such as consonant and dissonant intervals. They explained their finding as a consequence of greater efficiency in the discrimination of chords that deviate from conventional tonal categories than non-musicians. Specifically, a ‘mistuned’ chord was used which altered the middle note so that its pitch sounded between C and C sharp.

In the current study, we also expected to see neural evidence of a processing advantage for musically trained respondents perceiving microtonal intervals that are not prototypical, also set to sound between two equal-tempered tones, at a quarter-tone. We sought to elicit both overt response ratings and covert event-related brain potentials (ERPs), so as to compare subjective impressions of sounds (which might reflect any top-down influences) with the neurophysiological processing of the acoustic signal (which should reflect bottom-up features, especially at the early times after stimulus onset). Thus ERPs were preferred over other measures of brain response for their high temporal resolution [6]. Essentially, we adapted the procedures of Schön et al. [6] to address our questions concerning microtonality. Since the averaged ERP waveform collapses across several underlying latent components, the individual amplitudes and durations of these components can affect the form of the averaged ERP waveform [14]. Rather than concentrating on peaks in the signal [9–11], we adopted the approach to ERP data analysis of Schön et al. [6], analysing data in latency bands and inferring underlying components from these broader measures.

Based on the above, our core hypotheses for the experiment were:

1. Microtonal intervals are perceived differently from ET intervals.
2. The responses of musicians and non-musicians are distinct.
  More specifically:
3. Microtonal intervals will be less liked and more rough than ET intervals, in a pleasantness hierarchy: MT > Dissonant > Consonant.
4. ERP responses will distinguish MT and ET intervals.
5. The early ERP responses (predominantly perceptual/bottom-up) before 300ms will be larger for consonant > dissonant > MT intervals.
6. The subsequent ERP responses (reflecting cognitive processes to a greater extent) will be greater for musicians than non-musicians.

In short, our aim was to contrast overt and covert measures of listeners’ responses to unfamiliar microtonal intervals, to determine how these are perceived in comparison to more familiar 12-TET tunings. We also tested the hypothesis that musical training would differentially affect perceptions, finding this to be the case, with results suggesting that musical expertise facilitates the sensory and perceptual discrimination of microtonal intervals from 12-TET intervals, and an increased ability to categorize such intervals.

## Method

### Ethics statement

Ethical approval for this study was obtained from the University of Western Sydney Human Ethics Research Committee. Written, informed consent was obtained from participants prior to commencement of the session. Participants were paid 20AUD for travel expenses incurred in taking part in the study.

### Participants

A group of 20 adults (Mean age = 30.65 yrs, *SD* = 5.45; 8 female) were recruited through a convenience strategy. This group comprised equal numbers of musicians (*N* = 10, Mean age = 33.7 yrs, *SD* = 4.99; 3 female) and non-musicians (*N* = 10; Mean age = 27.6 yrs, *SD* = 4.14; 5 female). Musicians achieved a score greater than 500 (*M* = 925.2, *SD* = 97.96), and non-musicians a score less than 500 (*M* = 183.9, *SD* = 96.18), out of a possible 1000 on the Ollen Musical Sophistication Index questionnaire (OMSI, [15]). All participants were right-handed with the exception of one musician who was left-handed. No participant self-reported neurological conditions, or to be taking medication that could interfere with their cognitive abilities [16]. All participants self-reported normal, or corrected to normal, vision and normal hearing abilities.

### Stimuli

Two-note chords were performed on the Apple AUDLS piano 1 instrument. The lowest pitch in all chords was middle C, midinote 60. Chords were all 350ms long, in common with Itoh et al. [11,17], and performed at a velocity of 127 (the maximum) using MAX 4.6. Quarter tones were chosen so as to be within the critical bandwidth, and were achieved by pitchbend, where 16 up from normal 64(/127) MIDI-control value produced a quarter tone. See Table 1.

**Table 1:** Equal Tempered and Microtonal Stimulus Sets.

	Equal tempered set	Microtonal set
1	C/Db—minor 2^nd^	C/C ¼ tone sharp
2	C/D—major 2^nd^	C/C# ¼ tone sharp
3	C/Eb—minor 3^rd^	C/D ¼ tone sharp
4	C/E—major 3^rd^	C/Eb ¼ tone sharp
5	C/F—perfect 4^th^	C/E ¼ tone sharp
6	C/F#—diminished 5^th^	C/F ¼ tone sharp
7	C/G—perfect 5^th^	C/F# ¼ tone sharp
8	C/Ab—minor 6^th^	C/G ¼ tone sharp
9	C/A—major 6^th^	C/Ab ¼ tone sharp
10	C/Bb—minor 7^th^	C/A ¼ tone sharp
11	C/B—major 7^th^	C/Bb ¼ tone sharp

All of the two-note chords had a lowest pitch of middle C. Microtonal counterparts to the equal tempered intervals were sharpened by one quarter tone.

The chords varied little in their computational harmonicity (measured using the CNMAT MAXMSP external), but showed modest changes in computational roughness (measured using the Sonic Visualiser plugin by Jamie Bullock). There were no sequential tone pairs in this experiment: all were simultaneous.

The chords were recorded internally in the Mac using Wiretap Pro (16 bit, 44.1 kHz, aiff). Then they were imported into Audacity. There they were edited at a zoom at which 650ms of total file occupied the screen width, such that there was less than 3ms before the visual trace of the soundwave commenced and such that the whole sound file was very close to 500ms in length. Note that a 350ms activated tone is audible for a bit longer than 350ms. After editing, each sound was normalised in Audacity to -3dB (and DC offset). The last 50ms were faded out, and the stimulus was exported as a WAV.

Stimuli were presented using the Compumedics Neuroscan audio system controlled by Stim2 software. Audio was presented binaurally through ER-3A ABR insert earphones at a level of 80dB.

### Procedure

After giving written, informed consent, participants completed a short background demographic questionnaire. The experimental session was conducted in two parts.

#### ERP and roughness ratings

**Fig 1:**
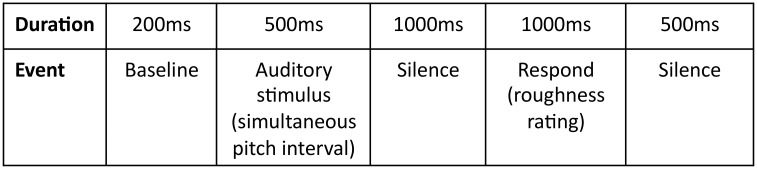
Schematic flow of each trial for ERP acquisition and roughness rating. The inter trial interval is 3200ms.

In the first part, ERP and subjective roughness responses were recorded successively. We measured perceived roughness to try to elicit more fine-grained consideration of the sounds particularly by the musician participants. Participants were presented with intervals in three test blocks containing 12 sets of 11 intervals. A training block, containing two sets of 11 intervals, preceded the three test blocks. Between each set of 11 intervals, stimulus presentation paused allowing participants a brief break. When they were ready to continue, participants pushed a button on the computer keyboard and the program advanced to the next set of 11 intervals. Each of the 11 intervals was presented for 500ms duration followed by 1000ms of silence. The instruction to indicate perceived roughness then appeared on the black computer screen (1000ms duration) followed by a further 500ms of silence. A baseline period of 200ms preceded each interval, or trial, making a total inter–trial–interval of 3200ms (Fig 1). With the exception of the response instruction, the computer screen appeared blank. Participants made their response as to how rough they perceived each interval to be on a five–point scale (very rough (1)—very smooth (5)) by depressing keys on a computer keyboard. Participants were asked to remain as still as possible to reduce movement contamination of the ERP trace. Their eyes remained fixed on the centre of the computer screen during each set of trials, and their fingers in contact with the response keys to minimize movement necessary to make their responses. Furthermore, the 1000ms silence (and stillness) between stimulus offset and response instruction in the trial aimed to reduce the possibility of motor planning and execution contaminating the ERP trace. Previous research has shown that the reaction time and the time taken to execute the movement response to a stimulus in an ERP paradigm takes approximately 600-900ms, depending on the complexity of the task [18,19]. Therefore, following the 1000ms silence in each trial, the 1000ms response period and additional 500ms silence would have provided participants sufficient time to complete their motor response and prepare for the next trial.

The number of 12-TET (perfect–consonant, imperfect–consonant, and dissonant) and microtonal intervals presented within the training and test blocks varied. The training phase contained an equal number of 12-TET and microtonal intervals. The intervals were randomised within the training block, but the order remained the same for all participants. The three test blocks following contained ratios of 12-TET to microtonal interval sets of 12:2, 6:6, and 2:12 respectively. Within these constraints, intervals were presented in a different random order for each participant, but the same interval was never presented twice in immediate succession. The ERP and roughness response part of the experimental session took place in an electrically shielded room.

Electroencephalographic (EEG) recordings were made using the Compumedics Neuroscan SynAmps2 system running Scan 4.4 software. Data were acquired through Ag-AgCl electrodes in a 64-channel cap from 28 Cephalic monopolar electrode sites (FP1, FP2, F7, F3, FZ, F4, F8, FC5, FC1, FC2, FC6, T7, C3, CZ, C4, T8, CP5, CP1, CP2, CP6, P7, P3, PZ, P4, P8, O1, OZ, O2), and the right mastoid (M2). Horizontal eye movements (HEOG) were recorded using bipolar electrodes, one adjacent to each eye’s external canthus. Vertical eye movements were recorded using a monopolar electrode beneath the left eye (VEOL). A forehead electrode served as the ground and the left mastoid (M1) as the reference. Data were recorded with a bandpass filter of DC-30Hz, and a sampling rate of 250Hz. Participants’ impedance values did not exceed 10kOhms.

#### Liking ratings

The ERP/roughness portion of the experiment was followed by the measurement of liking ratings of the same stimuli, gathered in a purely behavioural paradigm. These were similarly collected on a five-point scale (really dislike (1)–really like (5)) via computer keyboard. Liking was of interest since it is a response which does not require or encourage the use of special knowledge. This portion of the experiment was run on a MacBook using PsyScope X software. Sound was delivered binaurally through AKG headphones. This second part of the experimental session consisted of one test block containing 66 interval presentations (3 equal-tempered sets of 11 intervals plus 3 microtonal sets). Each interval was presented for 500ms duration followed by the instruction to make a response. Once participants had made their response, the program advanced to the next interval in the sequence. Within this test block, equal numbers of equal-temperament and microtonal intervals were presented in a different random order for each participant.

### Offline neurophysiological data analysis

A linked mastoid reference was derived offline between the left mastoid reference electrode (M1) and the right mastoid monopolar electrode (M2). A bipolar channel for vertical eye movements (VEOG) was created between the VEOL channel and the cephalic monopolar electrode above the left eye (FP1). Each participant’s data were bandpass filtered 0.01-30Hz with a 24dB per octave roll off. Blocks of data contaminated with movement were rejected. Eyeblink artefact was reduced using a spatial singular value decomposition method based on the VEOG channel. Responses were epoched around stimulus onset (-200 to 800ms). Baseline correction was performed based on the pre-stimulus interval, and automatic artefact rejection in the order of +/-65microvolts was applied. Each participant’s data were averaged in 100ms latency bands from stimulus onset to 600ms and data were exported for analysis. Data from three non-musicians and two musicians were excluded from analyses, owing to an inadequate signal to noise ratio.

### Data modelling

Multi-level linear models of the dependent variables (perceived roughness, liking, or ERP amplitude) were developed stepwise, testing the fixed effects of music theoretic interval category (consonant, dissonant, microtone), musical training category (musician, non-musician) and the random effects of musical experience (OMSI), subject and, for ERP analysis, electrode. Multi-level linear models [20] enhance the statistical reliability of primary fixed effect analyses by permitting assessment of inter-individual (and if necessary inter-item or inter-token) variation (the so-called random effects). Here we considered inter-individual variation, and could assess distributions across individuals in relation both to the intercepts of responses, and to the slope in response to any independent variable. We also used random effect determination in relation to the 26 cephalic electrodes included in the analyses (which omitted Fp1 and Fp2). Model selection used the Bayesian Information Criterion (BIC) and likelihood ratios to determine the most parsimonious fit, and was done in R using the lme4 package. β represents the fixed effect coefficient on the predictor being considered, which is presented below with reference to the specified base level of the factor, e.g. dissonant or microtonal versus the base level consonant.

## Results

### Behavioural ratings

#### Roughness

**Fig 2:**
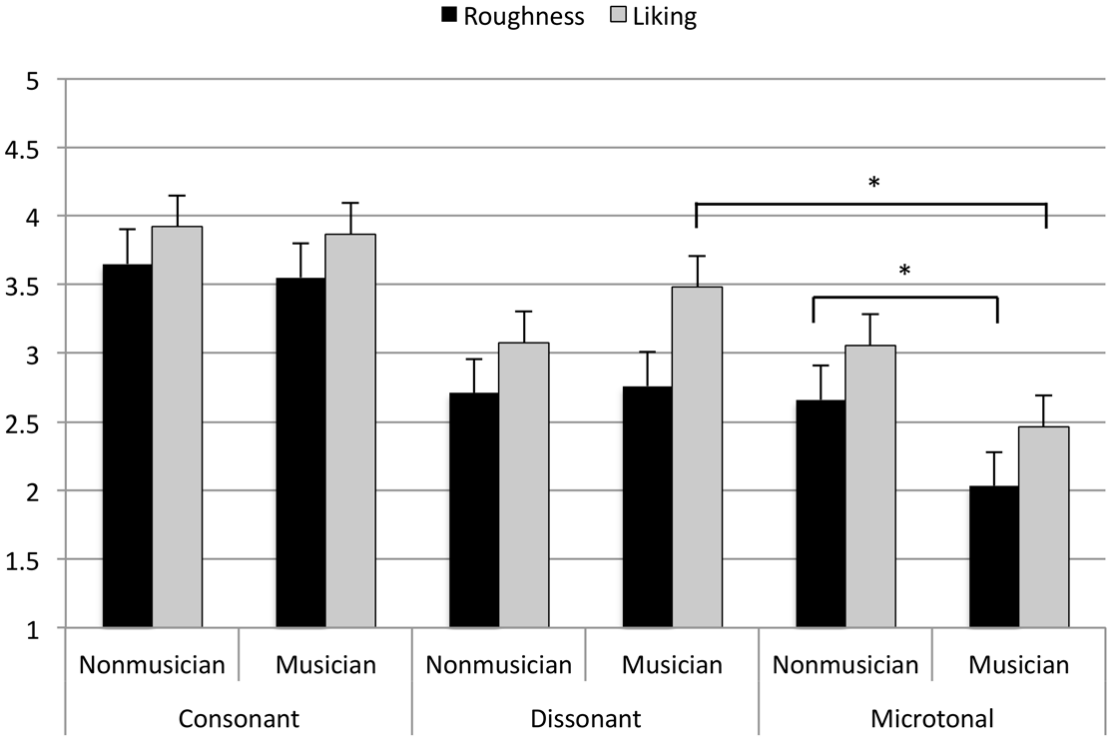
Mean perceived roughness and liking ratings of consonant, dissonant, and microtonal intervals by musicians and non-musicians. For roughness, a rating of ‘1’ represents ‘very rough’ and ‘5’ represents ‘very smooth’. For liking, a rating of ‘1’ represents ‘really dislike’ and ‘5’ represents ‘really like’. Asterisks indicate a significant result.

Perceived roughness was significantly greater for microtonal intervals than for dissonant intervals (β = 0.73, *t*(8) = -26.29, *p* < .0001), and for dissonant intervals than for consonant intervals (β = 0.79, *t*(8) = 28.51, *p* < .0001). Fig 2 illustrates a significant interaction between interval category and music training group, whereby musically trained respondents rated microtonal intervals as more rough than non-musicians (where a rating of ‘1’ = ‘very rough’, and ‘5’ = ‘very smooth’). Musical experience contributed to the optimal model of perceived roughness ratings (random intercept, *SD* = 0.35) but taking into account the random effect of individual participant did not. Surprisingly, neither musicians’ nor non-musicians’ ratings of perceived roughness correlated with a physical measure of the roughness of the stimuli (Musicians, *r* = -.01; Non-musicians, *r* = -.16).

#### Liking

Liking ratings were significantly greater for consonant intervals than for dissonant intervals (β = 0.38, *t*(8) = 12.15, *p* < .0001) and for dissonant intervals than for microtonal intervals (β = 1.02, *t*(8) = -32.14, *p* < .0001). Fig 2 illustrates a significant interaction between interval category and musical training group, whereby musically trained respondents disliked microtonal intervals significantly more than dissonant intervals, while non-musicians reported a similar degree of liking for these two interval types. Individual participants contributed to the optimal model of liking ratings as a random effect, (random intercept, *SD* = 0.41), but musical experience did not.

In summary, music theoretic distinctions between the intervals used were meaningfully related to differences in the ratings of roughness and liking. In addition, musical experience appears to have mediated the perception of differences between dissonant and microtone intervals, with non-musicians giving similar ratings for each, and musicians preferring dissonant over the less commonly used microtonal intervals, rating them as less rough.

### ERP

#### Music theory

The music theoretic category of the intervals predicted the amplitude of ERPs at all time windows, with a significant difference in the impact of interval consonance occurring throughout the signal except during the 304-400ms time window. At all of these windows, response amplitude was significantly greater for consonant intervals than other intervals. In some time windows, microtonal intervals produced distinct responses both from those to consonant and from those to dissonant 12-TET intervals, consistent with our core hypothesis.

Table 2 sets out the fixed and random effects for the best-fit model of ERP amplitude at each time window. Response to dissonant and microtonal intervals is characterised by a reversal of their relative negativity partway through the signal. During the first half of the response, dissonant intervals are intermediate between consonant and microtone stimuli, while during the second half of the response this order is reversed.

**Table 2:** Fixed and Random Effects for the Best-fit Model for each Window of the ERP.

Time window	Fixed effects in model	Fixed effect magnitude and significance	Random effects
4-100ms	Music theory	consonant > microtonal (β = 0.33, *t*(11) = -4.87, *p* = .0005)	Subject (slope)
			Electrode (intercept)
104-200ms	Music theory	consonant > dissonant (β = 0.49, *t*(14) = 2.556, *p* = .02)	Subject (slope)
		consonant> microtonal (β = 0.54, *t*(14) = -3.57, *p* = .003)	Electrode (slope)
	Music training	n.s.^a^	
204-300ms	Music theory	consonant> microtonal (β = 0.84, *t*(16) = -4.814, *p* = .0002)	OMSI (slope)
		dissonant > microtonal (β = 0.68, *t*(16) = -2.157, *p* = .05)	Electrode (slope)
	Music training	n.s.	
	Music theory*training	n.s.	
304-400ms	Music theory	n.s.	Subject (intercept)
	Music training	musicians > non-musicians (β = 1.002, *t*(14) = -2.48, *p* = .02)	Electrode (slope)
404-500ms	Music theory	consonant > dissonant (β = 0.56, *t*(16) = 2.513, *p* = .02)	Subject (slope)
	Music training	n.s.	Electrode (slope)
	Music theory*training	n.s.	
504-600ms	Music theory	consonant > dissonant (β = 0.67, *t*(16) = 4.058, *p* = .001)	Subject (slope)
			Electrode (slope)

β coefficients are expressed as absolute values (i.e. without the sign).

^a^n.s. = not significant.

#### Music training

**Fig 3:**
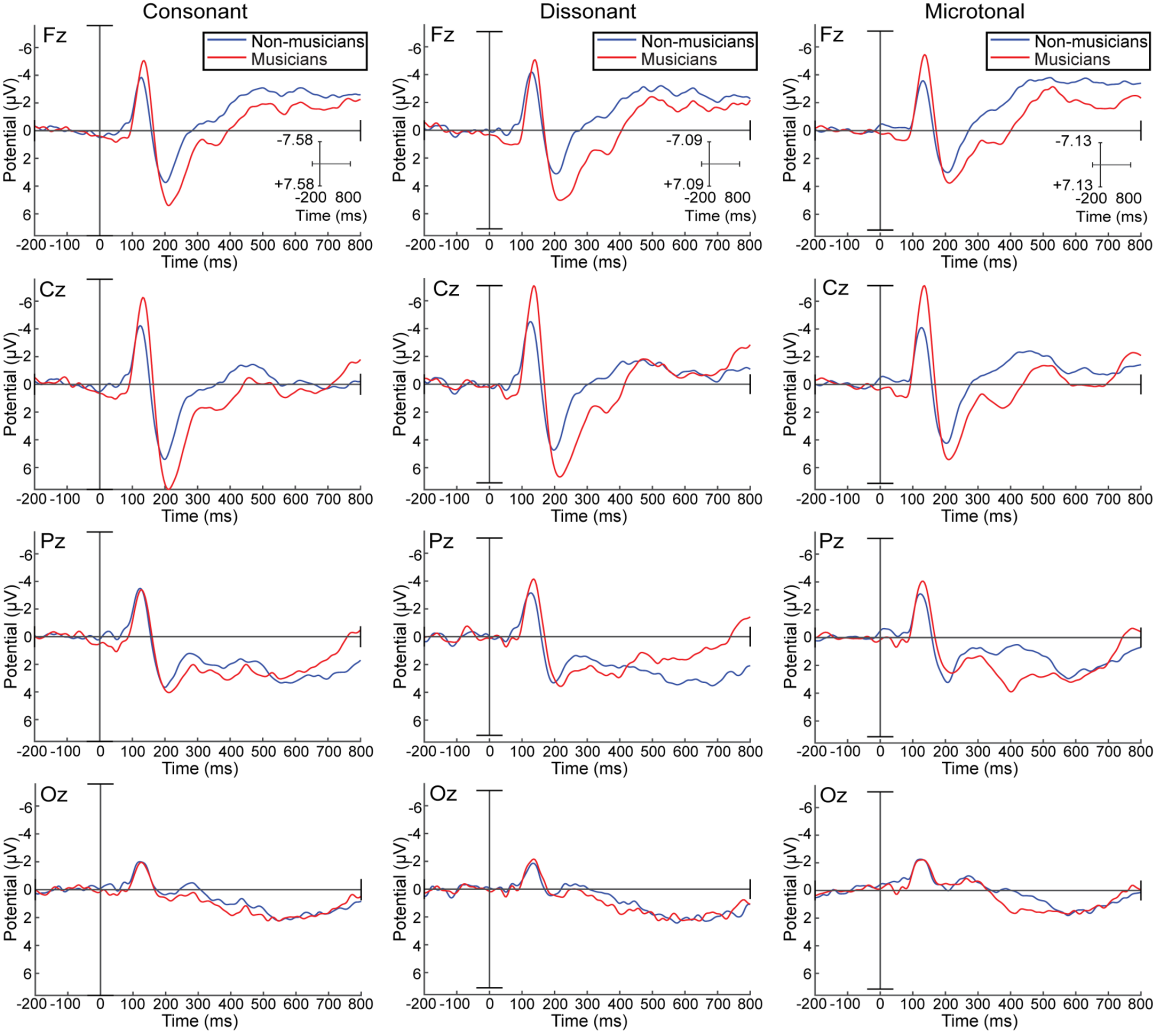
Average ERPs for consonant, dissonant and microtonal intervals for musicians and non-musicians. Musician (red line) and Non-musician (blue line) group average ERPs at four midline sites (FZ, CZ, PZ, and OZ) for consonant, dissonant, and microtonal intervals.

Including music training category (musician vs. non-musician) improved all the models of ERP amplitude between 104 and 500ms, but not those for the earliest or latest windows of the response. However, a statistically significant effect of music training was only evident between 304-400ms (see Table 2), when the ERP of the musicians showed more positivity than that of the non-musician group (Fig 3). It is noteworthy that the individual measure of musical experience as measured by the OMSI only improved the model between 404-500ms (Table 2), suggesting that group category (musician vs. non-musician) and individual musical experience bear a different relationship to the ERP.

#### The interaction between music theory and music experience

**Fig 4:**
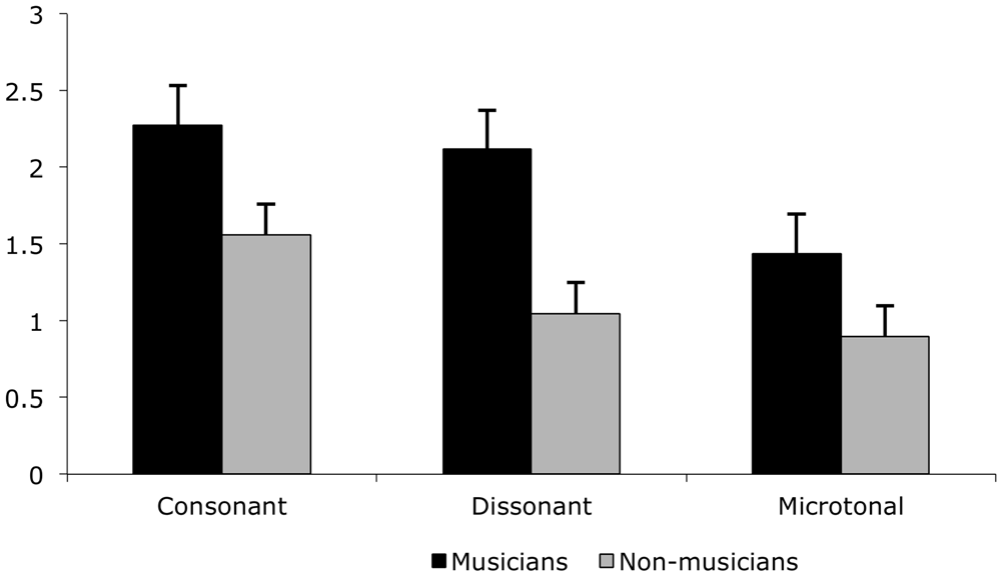
Mean ERP amplitude for musicians and non-musicians for consonant, dissonant and microtonal intervals between 204-300ms. Mean amplitude, and standard error, for the three music theoretic stimulus categories for musicians and non-musicians between 204-300ms.

**Fig 5:**
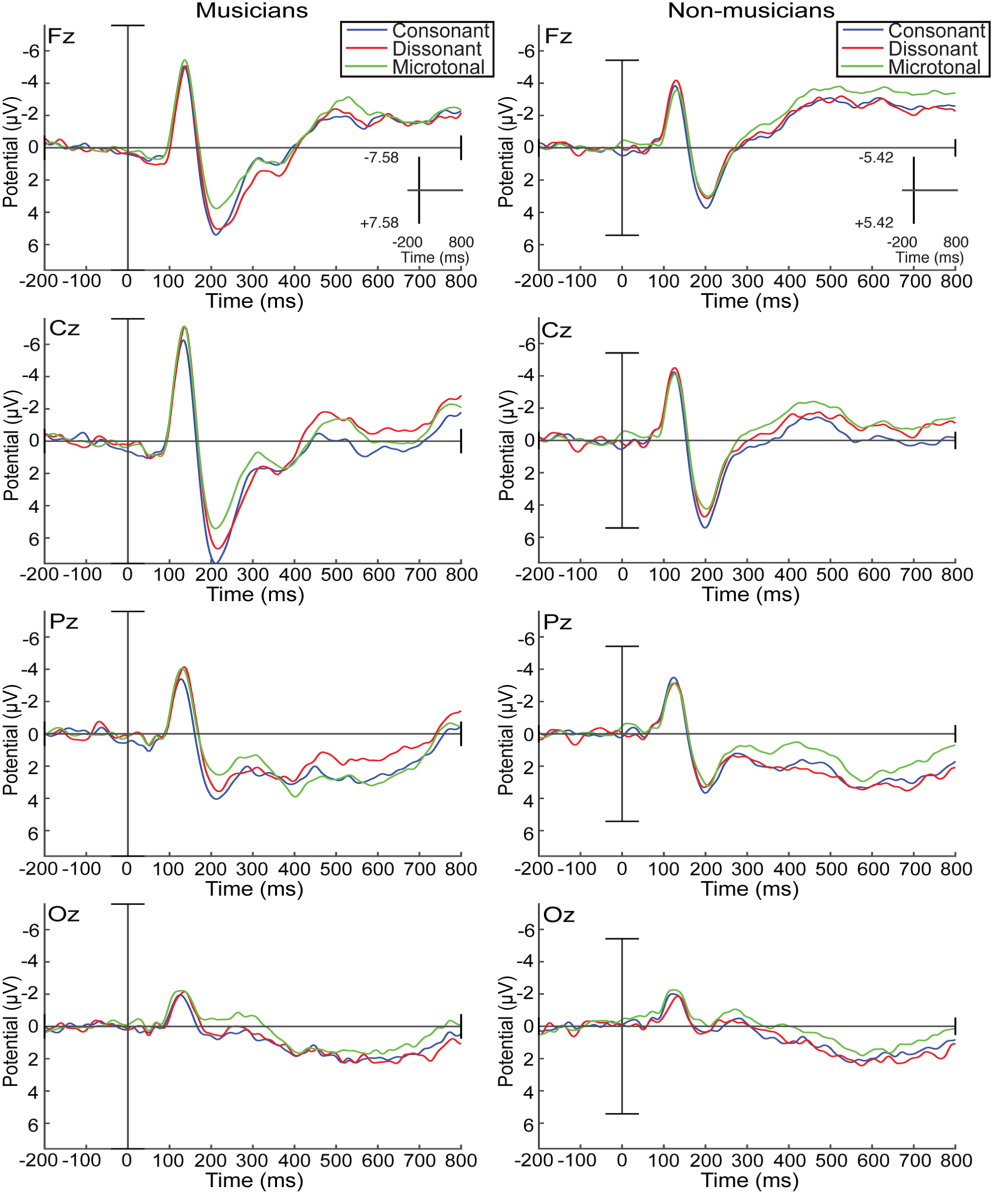
Musician and non-musician average ERPs for consonant, dissonant and microtonal intervals. Musician and Non-musician ERPs at four midline sites (FZ, CZ, PZ and OZ) for consonant intervals (blue line), dissonant intervals (red line), and microtonal intervals (green line).

An interaction between music theory and music training category contributed to the best fit models of the ERP for 204-300ms, and 404-500ms. For musicians, the P2-N2 complex corresponding to the 204-300ms window shows lesser positivity for microtonal intervals than for the 12-TET intervals. This is accompanied by a decrease in amplitude (Figs 4 and 5).

**Fig 6:**
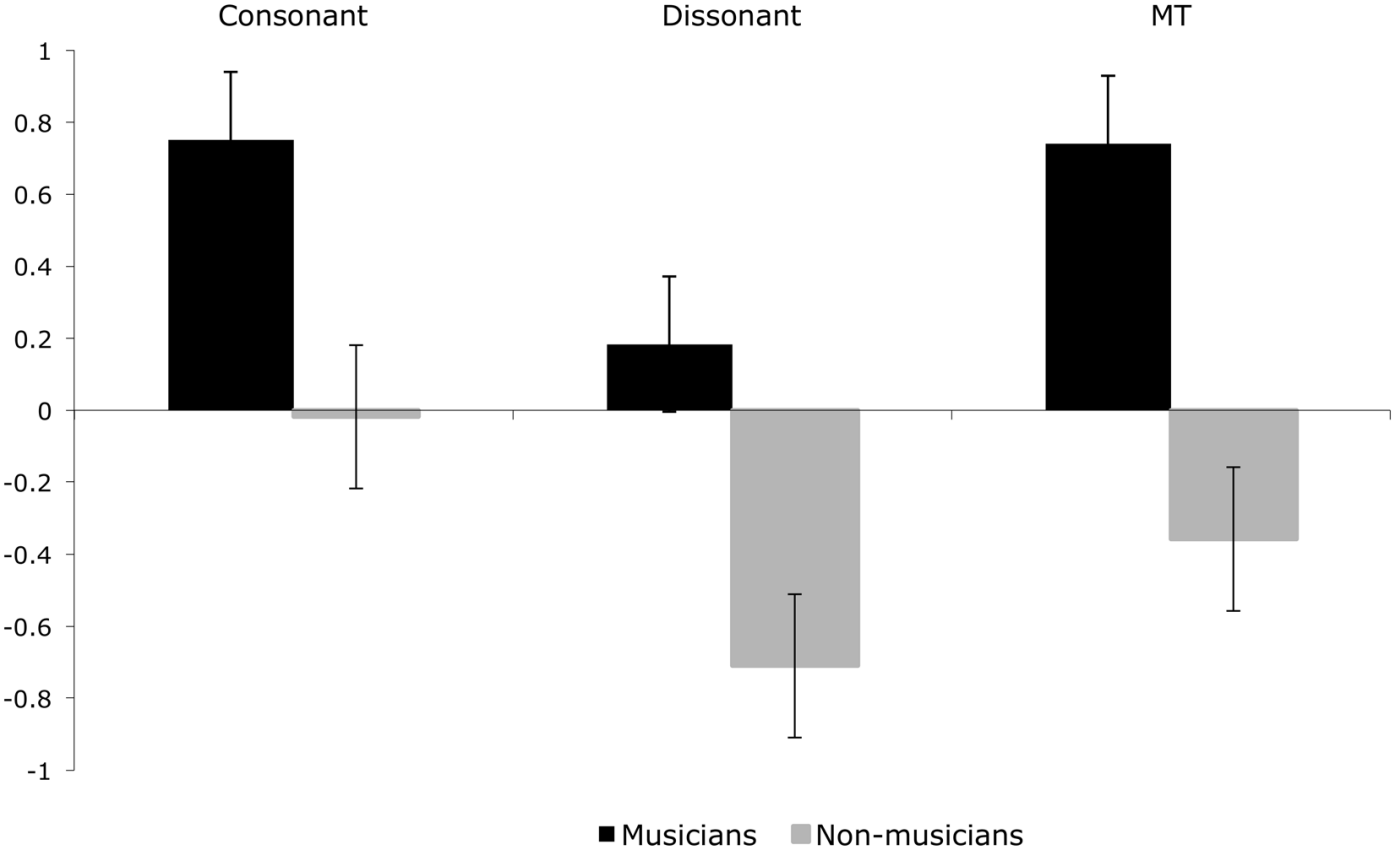
Mean ERP amplitude for musicians and non-musicians for consonant, dissonant and microtonal intervals between 404-500ms. Mean amplitude, and standard error, for the three music theoretic stimulus categories for musicians and non-musicians between 404-500ms.

Fig 6 illustrates different degrees of negativity between musician and non-musician respondents for each of the music theoretic categories in the 404-500ms window, possibly reflecting a negative N4 component associated with difficulties in stimulus categorisation; functionally similar to the N2 component, but with a delayed latency [6]. The greater negativity for all interval types for the non-musicians might suggest a greater cognitive load than for musicians, who exhibited the greatest negativity on presentation of dissonant intervals.

While musicians’ P2 showed the smallest amplitude for microtone intervals compared to 12-TET consonant or dissonant intervals, perhaps indicating difficulty in sensory discrimination, the high positivity at the time of the later N4 component suggests that microtone intervals were easy to categorise at the cognitive level. Ease of categorisation appears to be on a par with consonant intervals for which musicians would have a strong sensory memory. Overall, our results show that for non-musicians, dissonant intervals were perhaps difficult to discriminate at the sensory level, and to categorise at the cognitive level. Interestingly, non-musicians’ N4 for microtone intervals suggests that these intervals were easier to categorise than dissonant intervals, but not consonant intervals.

## Discussion

It has been hypothesized that simple integer ratios, such as characterize the consonant interval stimuli used in this study, facilitate both processing and storage in sensory memory [21]. Such perceptual fluency is often associated with liking [22] and should be reflected at a neural level. Behavioural and brain responses to the stimuli align to show that equal-tempered intervals were preferred (liked better) over the culturally less familiar microtonal intervals, also being perceived as less rough and eliciting a greater ERP amplitude (cf. [10]). As the interval ratio complexity increased from consonant through dissonant to microtonal intervals, perceived roughness increased, liking decreased, and the amplitude of the ERP decreased (though the difference between the mean ERP amplitude for dissonant and microtone intervals was only significant from 204-300ms—approximating the P2 component).

The musical experience of participants related to their judgements of interval roughness (as a random intercept in the model of the subjective rating data), and also to their pattern of ERP response (with musicians exhibiting greater negativity than non-musicians in the early response, but vice versa for later components). In the later portion of the ERP associated with more cognitive than sensory processing, between 304-400ms (approximating a P3 response), musicians exhibited a significantly more positive signal than their non-musician counterparts [9].

Interactions between the musical training of the respondents and their perceptions of the intervals were apparent both in terms of subjective ratings of the stimuli (roughness and liking) and ERP. Non-musicians perceived the roughness of dissonant and microtonal intervals to be equivalent, (dis)liking them equally, and obtained a similar magnitude ERP for these two interval types at the 204-300ms (P2) latency, while musicians perceived greater roughness for the microtonal intervals, liked them less, and obtained a smaller mean ERP amplitude at this time. Music culture inevitably has a bearing on the subjective ratings of music intervals with respect to liking, pleasantness, and perceived roughness. Western classical musicians have been trained to favour consonance, but also to understand and utilize dissonance, which surely impacted on their subjective ratings. While non-musicians gave similar ratings for both dissonant and microtonal intervals, musicians preferred dissonant over microtonal intervals, rating them as less rough.

It seems that while microtonal intervals are rated as more rough and less liked than dissonant intervals, non-musicians exhibited neural responses indicating processing demands similar to those associated with categorising dissonant intervals between 204 and 300ms. A possible explanation is that non-musician listeners aligned the microtone intervals with the nearest dissonant interval a quarter-tone away. In other words, microtone intervals were possibly categorically perceived as standard intervals. The psychological reality of microtonal intervals has been questioned [23], and it might be that the quarter-tone intervals employed in the current study were perceived categorically by respondents without musical training as belonging to an equal-tempered neighbouring interval “connected by the simplest possible just ratios” [23]. One alternative account for the lack of discernible difference in ERP between dissonant and microtone intervals by non-musicians at this time (204-300ms) is that of a floor effect.

## Conclusions

We found evidence that microtonal intervals are perceived differently from equal-tempered intervals. Subjective ratings of the roughness and liking of the intervals showed a behavioural difference between perceptions of microtonal and consonant intervals, with microtonal intervals rated as significantly more rough and less liked than consonant intervals. A measure of pre-conscious evoked response potentials to our equal-tempered and microtonal stimuli further confirmed distinct perceptions of these two categories of interval, with stronger amplitude ERP for equal-tempered than microtonal stimuli.

Differences between musicians and non-musicians in the perception of equal-tempered and microtonal intervals were apparent in ERPs. Two different response trajectories from early sensory through to later cognitive components of the signal can be traced. Initially, musicians show a sensory discrimination of microtonal intervals while non-musicians do not, followed by a later equalizing of the amplitude response to microtonal and consonant intervals, perhaps suggesting that microtonal stimuli were just as easily categorized as consonant stimuli by this group [6]. Overall, it seems that musical expertise facilitates the sensory and perceptual discrimination of microtonal intervals from equal-tempered intervals, and an increased ability to categorize such intervals, while non-musicians appear to have categorically perceived microtonal intervals as instances of their neighbouring equal-tempered counterparts. This interpretation would cohere with the results of the preliminary experiment, in which MT intervals were also treated by non-musicians in rather the same way they treat 12-TET intervals.
